# Concomitant early gallbladder carcinoma with primary sarcomatoid hepatocellular carcinoma: A case report

**DOI:** 10.3892/ol.2013.1288

**Published:** 2013-04-03

**Authors:** DONG XUE, KAI ZUO, XINJUN LI, HONGQIANG CHEN, YUNFEI XU, YU CHENG, YUXIN CHEN

**Affiliations:** 1 Department of General Surgery, Qilu Hospital, Shandong University, Ji’nan, Shandong 250012;; 2 Departments of General Surgery, The People’s Hospital of Binzhou, Binzhou, Shandong 256610, P.R. China; 3 Infectious Diseases, The People’s Hospital of Binzhou, Binzhou, Shandong 256610, P.R. China; 4 Pathology, The People’s Hospital of Binzhou, Binzhou, Shandong 256610, P.R. China

**Keywords:** sarcomatoid hepatocellular carcinoma, gallbladder carcinoma, computed tomography

## Abstract

Concomitant primary sarcomatoid hepatocellular carcinoma (SHC) with gallbladder carcinoma is a rare type of hepatobillary disease. To the best of our knowledge, this coexistence has rarely been reported. An 80-year-old male presented with right-sided epigastric pain and a low fever. Computed tomography (CT) imaging revealed a hypodense lesion in the right lobe of the liver and a regular intraluminal polypoid mass in the gallbladder. The patient underwent a partial hepatectomy of the right lobe of the liver and a cholecystectomy. Following pathological examination, the patient was diagnosed with SHC combined with gallbladder adenocarcinoma. The patient and his family refused post-operative adjuvant chemotherapy and radiation therapy. The patient succumbed to intrahepatic and lung metastases at six months post-surgery. In conclusion, concomitant gallbladder carcinoma and SHC may occur. Surgery-based multimodal treatment is the preferred strategy for compound tumors. Adjuvant chemotherapy or radiotherapy may be necessary for the high risk hepatobiliary malignancies.

## Introduction

Sarcomatoid carcinoma is a rare, aggressive malignancy containing mesenchymal and epithelial components. Sarcomatoid hepatocellular carcinoma (SHC) is an uncommon malignant lesion of the liver, with an incidence of 2% in surgically-resected cases and 4–9% in autopsied cases (1–4). Forming a diagnosis may be difficult due to its atypical presentation. Concomitant gallbladder carcinoma with primary SHC is a rare type of hepatobiliary disease that contains two different malignant lesions. To the best of our knowledge, such a case has not previously been reported. The present study reports a case of gallbladder carcinoma accompanied with SHC in a Chinese male. The management of this rare disease is also discussed. Written informed consent was obtained from the patient’s family for publication of the case report and accompanying images.

## Case report

**Figure 1:**
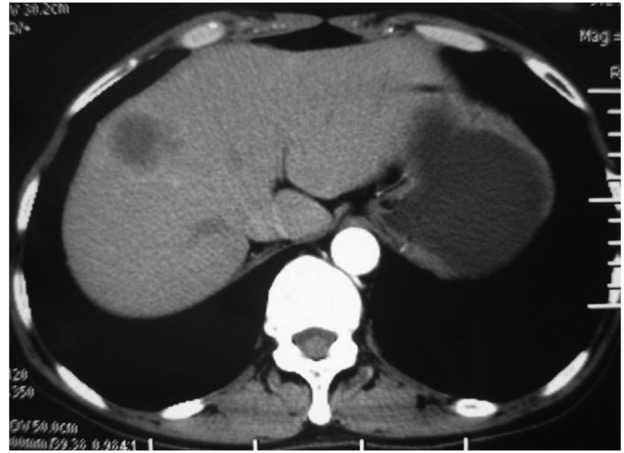
Abdominal computed tomography (CT) showing a hypodense lesion in the right lobe of the liver.

**Figure 2:**
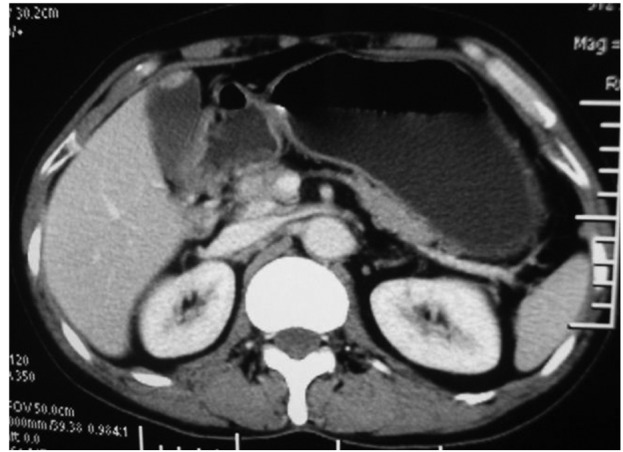
Abdominal computed tomography (CT) showing a regular intraluminal polypoid mass in the gallbladder.

**Figure 3:**
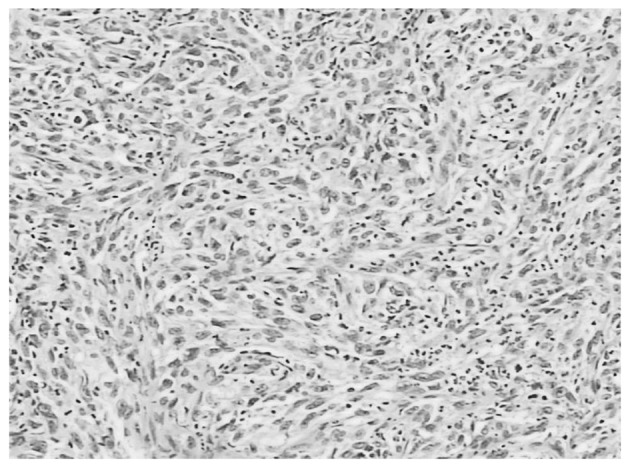
Histopathological image of SHC consisting of atypical, round spindle-shaped cells with large nuclei. (Hematoxylin and eosin staining; magnification, ×200). SHC, sarcomatoid hepatocellular carcinoma.

An 80-year-old male, who had been a hepatitis B virus (HBV) carrier for 10 years, was admitted to Qilu Hospital, Shandong University, Ji’nan, Shandong, China due to right-sided epigastric pain and a low fever occurring over the preceding 2 months. Additionally, there was no apparent loss in body weight and the routine blood tests were normal. The levels of tumor markers, including that of α-fetoprotein (AFP), carbohydrate antigen (CA) 19-9, CA 125, carcinoembryonic antigen (CEA) and CA 50, were all within the normal ranges. The liver function grade of the patient was Child-Pugh grade A. Contrast-enhanced computed tomography (CT) of the abdomen revealed a hypodense lesion consisting of a 5×4×3 cm mass in the right lobe of the liver (Fig. 1). Abdominal CT showed a regular intraluminal polypoid mass in the gallbladder with no demonstrable lymph nodes (LNs) in the pericholecystic or upper abdominal regions (Fig. 2). No evidence of intra-abdominal metastatic spread was observed. The first presumptive diagnosis was of hepatocellular carcinoma (HCC) and gallbladder adenoma, and the second was of gallbladder carcinoma with liver metastasis. Following a partial hepatectomy of the right lobe of the liver and a cholecystectomy, the patient was diagnosed with SHC combined with adenocarcinoma of the gallbladder *in situ* by pathological examination. On gross examination, the mass of the liver measured 5×3.5×3 cm. A papillary tumor of 12×10×7 mm was identified in the middle third of the gallbladder, with a basal portion of 7 mm. The surface cut appeared brown in the peripheral portion with a hemorrhage foci and massive necrosis in the center. On pathological examination, the tumor of the liver was composed of spindle-shaped tumor cells, resembling true sarcomas (Fig. 3). Histologically, the mass in the gallbladder was found to be a high-grade adenocarcinoma. The cancer stage of the patient was classified as type T1N0M0 using the classification of the American Joint Committee on Cancer (AJCC) (5). Immunohistochemically, the neoplastic cells were strongly positive for CK, CK2, CK7 and vimentin, showing evidence of their epithelial malignancy rather than a combination of HCC and sarcoma. Diagnosis of this condition may prove difficult as the histological features may mimic sarcoma. In view of the clinical history and the morphological and immunohistochemical observations, a diagnosis of primary SHC was made.

The patient recovered from the surgery with no complications, however, post-operative adjuvant chemotherapy and radiation therapy were refused. The patient developed progressive intrahepatic and lung metastases and finally succumbed to this six months post-surgery.

## Discussion

Patients with SHC often present with a poor prognosis due to skin, pleural and pelvic skeletal metastases (6–8). The incidence of SHC is highest in the fifth and sixth decades of life. The majority of cases are symptomatic with pain in the right upper quadrant of the abdomen. Transarterial chemotherapy, chemoembolization or radiofrequency ablation are presumed to promote sarcomatous changes in HCC (9).

The terminology and pathogenesis of SHC remain controversial issues. In the present study, the sarcomatous elements in the neoplasms were stained positive for the epithelial markers, as confirmed by immunohistochemical studies, which supported the pathological diagnosis of sarcomatoid carcinoma rather than carcinosarcoma. The pathogenesis of SHC has not yet been clarified due to the ongoing debate on whether SHC is derived from the transition of an ordinary HCC to a sarcomatous HCC, or if it is a double cancer of HCC and hepatic sarcoma. One possible origin of sarcomatoid carcinoma is the collision theory of independent neoplasm growths from multipotent stem cells, epithelial to mesenchymal transition, and the other possible origin is the combination of the two tumors (2,3,10). The majority of investigators consider that the sarcomatous component is derived from a dedifferentiation of anaplasia from an ordinary HCC. It is difficult to gauge if there is a connection between SHC and HBV, however, 54% of patients are HBV carriers (11).

The treatment of choice for SHC is surgical resection. The utility of other treatments, including chemotherapy, radiotherapy and immunotherapy, remains unproven. The formation of a clinical diagnosis is difficult and relies on a combination of imaging techniques, including ultrasound, CT scans and even pathological techniques, such as fine-needle aspirations with biopsies of the suspicious mass. The expression of cytokeratin by the spindle cell component of the sarcomatoid carcinoma, as observed by immunohistochemistry, suggests a common origin rather than a collision tumor composed of sarcoma and carcinoma (2,3). There were no transitional zones between the carcinomatous and sarcomatous components in the present study, which did not support the transformation theory. Furthermore, the sarcomatous elements were stained positive for the epithelial markers that had been applied for immunohistochemical staining, which supported the pathological diagnosis of sarcomatoid carcinoma rather than carcinosarcoma.

SHC usually presents as a large mass with peripheral enhancement, central necrosis, variable enhancement of the solid portion with or without a tumor capsule, and intrahepatic metastasis (11). When gallbladder carcinoma is accompanied by minor SHC, it is not difficult to deal with the malignancy and early gallbladder lesions at the same time, as the preservation of enough liver parenchyma is easy to achieve, resulting in a successful surgery. Adjuvant chemotherapy and/or radiation therapy may be necessary for the patient following the resection. Intrahepatic metastasis and adjacent organ invasion are relatively more common with SHC than with ordinary HCC (10).

In conclusion, SHC is a relatively rare, malignant hepatic neoplasm. Although it is not easy to speculate on the coexistence of gallbladder adenocarcinoma and SHC, pre- and post-operative diagnoses may be essential. Surgical involvement is the mainstay of therapy. Further studies are essential to expound the possible associations involved. The prognosis of patients with multiple primary carcinomas is extremely poor.
